# Mechanistic Study on the Alleviation of Endometritis in Mice Through Inhibition of NF-κB and MAPK Signaling Pathways by Berberine and Carvacrol

**DOI:** 10.3390/microorganisms13051051

**Published:** 2025-04-30

**Authors:** Xiaoshan Liang, Yabo Wang, Tianyi Li, Peilong Li, Guojun Jiang

**Affiliations:** College of Veterinary Medicine, Hebei Agricultural University, Baoding 071000, China

**Keywords:** endometritis, mice, berberine, carvacrol, NF-κB, MAPK

## Abstract

Berberine and carvacrol have demonstrated anti-inflammatory effects; however, their therapeutic potential in endometritis remains unclear. (Aims) This study aimed to examine the anti-inflammatory properties of berberine and carvacrol in a murine model of endometritis, with a focus on the underlying molecular mechanisms. (Main methods) The model was established via vaginal instillation of 0.1 mL of a mixture containing *Escherichia coli*, *Staphylococcus aureus*, and *Group B Streptococcus*, followed by treatment with 0.1 mL of berberine (4 mg/mL) and carvacrol (0.125 mg/mL) six days post-infection. All mice were euthanized on day 13, and uterine tissues were collected for subsequent analyses. (Key findings) Treatment with berberine and carvacrol significantly reduced tissue injury associated with endometritis, decreased mRNA expression of TLR2 and TLR4 (*p* < 0.01), and inhibited the phosphorylation of NF-κB and MAPK pathway-associated proteins, as well as the mRNA expression and levels of pro-inflammatory cytokines. (Significance) Berberine and carvacrol exhibit significant therapeutic effects against bacterial-induced endometritis by reducing TLR2 and TLR4 expression, inhibiting NF-κB and MAPK pathway activation, and decreasing pro-inflammatory cytokine production, thus demonstrating robust anti-inflammatory activity.

## 1. Introduction

Endometritis is a common clinical disorder in dairy cows, characterized by fever, anorexia, reduced milk production, purulent uterine discharge, and uterine abnormalities detectable via rectal examination. It primarily arises from microbial infections, predominantly bacterial pathogens such as *Escherichia coli*, *Streptococcus pyogenes*, *Fusobacterium necrophorum*, and *Staphylococcus aureus* [1,2]. Pathogens can enter the uterus through the vagina via the open cervical canal during production processes, insemination, or uterine examinations, leading to intrauterine infections. These infections trigger endometritis, disrupt the uterine lining, impair follicular function, and affect oocyte development, resulting in infertility in dairy cows [3]. Endometritis in dairy cows has been widely reported across the globe; an epidemiological study in Hebei Province revealed a total incidence of 24.62%, peaking at 43.08% in July. This underscores the significant impact of endometritis on the health of the dairy industry [4,5]. Clinically, antibiotics (administered intrauterinely or systemically) and prostaglandins (PGF2α, systemically) are commonly used to treat or prevent endometritis in dairy cows [6]. However, *E. coli* has demonstrated high resistance to multiple antibiotics, likely due to the extensive use of broad-spectrum antibiotics in clinical treatment [7]. Consequently, there is an urgent need for new therapeutic strategies that can effectively combat bacterial endometritis, particularly for drug-resistant pathogens.

Chinese medicines exhibit a broad spectrum of antibacterial and anti-inflammatory properties. Berberine and carvacrol, two active components derived from Chinese medicine, possess multiple effects, including antibacterial, antioxidant, anti-inflammatory, and immune-enhancing properties. Both compounds are hydrophobic and can interact with the lipid bilayer of bacterial membranes, destabilizing them and exerting antibacterial effects [8]. Silva Lima demonstrated the use of trace amounts of carvacrol to treat inflammatory edema in the rat foot, reducing levels of TNF-α and IL-1β [9]. NF-κB (nuclear factor κB) is a family of transcription factors widely expressed in cells, composed of hetero- or homodimers formed from subunits including p50, p52, p65, c-Rel, and RelB; activation occurs in response to inflammatory mediators, pathogen-associated molecular patterns, or damage-associated molecular patterns through membrane receptors such as Toll-like receptors (TLRs) and tumor necrosis factor receptors (TNFRs), leading to rapid induction of pro-inflammatory gene expression. The mitogen-activated protein kinase (MAPK) signaling pathway comprises three main cascades: extracellular signal-regulated kinase (ERK), c-Jun N-terminal kinase (JNK), and p38 MAPK. These pathways regulate cellular processes including proliferation, differentiation, stress responses, apoptosis, and inflammation. Synergistic interactions between NF-κB and MAPK pathways amplify inflammatory responses and enhance the transcriptional activation of pro-inflammatory genes such as interleukin-8 (IL-8) [10]. Pretreatment with carvacrol inhibits ERK 1/2 phosphorylation, which inhibits the activation of the NF-κB signaling pathway and attenuates lipopolysaccharide (LPS)-induced inflammatory response [11]. In contrast, berberine targets the eukaryotic translation initiation factor 2 alpha kinase 2 (EIF2AK2), modulating multiple signaling pathways and alleviating inflammation by downregulating the expression of IL-18β, IL-2, and TNF-α [12]. However, the therapeutic potential of berberine and carvacrol in the treatment of endometritis remains underexplored. Therefore, this study aimed to construct a murine model of endometritis induced by mixed bacterial infection and investigate the therapeutic mechanisms of these herbal components, thereby laying the groundwork for the development of novel alternative antibiotics for the prevention and treatment of endometritis.

## 2. Materials and Methods

### 2.1. Drugs and Reagents

The 64 8-week-old female Kunming mice used in the experiment were purchased from Spearfish Biotechnology Co. (Beijing, China). This study followed the NIH Guide for the Care and Use of Laboratory Animals. Berberine (CAS:2086-83-1) was purchased from Hubei Jusheng Technology Co. Ltd. (Tianmen, China); carvacrol (CAS:499-75-2) was purchased from Shanghai Macklin Biochemical Co. (Shanghai, China); *Escherichia coli* (1919D3) was provided by the College of Animal Medicine, Hebei Agricultural University; *Staphylococcus aureus* (186158) and *GBS* (NBCC 336970) were purchased from Guangzhou Leizhi Biotechnology Co. Ltd. Guangzhou, China); ELISA kits (TNF-α (ml002095), IL-1β (ml107159), IL-6 (mI098430), and IL-8 (ml063162)) were purchased from Shanghai Enzyme-linked Biotechnology Co., Ltd. (Shanghai, China); FastKing gDNA Dispelling RT Super Mix (FP313) was obtained from Stiangen Biotech (Beijing, China) Co., Ltd.; SuperReal PreMix Plus (FP205) was purchased from Tiangen Biotech (Beijing, China) Co., Ltd.; ECL chemiluminescence kit was purchased from New Cell & Molecular Biotech Co. (Suzhou, China); rabbit-derived anti-mouse primary antibody (including rabbit β-Actin primary antibody (bs-0061R), rabbit p-IκB-α primary antibody (bs-2513R), rabbit p-p65 primary antibody (bs-0465R), rabbit p-ERK1/2 primary antibody (bs-1020R), rabbit p-JNK1 primary antibody (bsm-61084R), rabbit p-p38 primary antibody (bsm-55529R)) and rabbit secondary antibodies (IHC005) were purchased from Beijing Biosynthesis Biotechnology Co. (Beijing, China).

### 2.2. In Vitro Bacteriostatic Assay of Berberine and Carvacrol

The minimum inhibitory concentration (MIC) and minimum bactericidal concentration (MBC) of berberine and carvacrol were determined using the culture broth dilution method (CLSI M07-A8). The MIC was selected as the subsequent test drug concentration.

### 2.3. Drug Safety Tests of Berberine and Carvacrol

Drug irritation was detected using the acute eye irritation test [13]. The eyes of the rabbits were injected with berberine and carvacrol, and the damage and recovery of the conjunctiva, iris, and cornea were observed; using the accumulation toxicity test, the mice were injected with berberine and carvacrol vaginally and then euthanized 24 h later; the intact uteri were removed by dissecting the uterus. Any congestion and edema were observed with the naked eye, and histopathological changes were observed with a microscope. According to the results of the drug safety test, the Chinese medicine components with a low bacterial inhibitory concentration and which were safe for mice uteri were selected for the subsequent therapeutic test.

### 2.4. Modeling of Bacterial Infectious Endometritis

A total of 10^8^ CFU/mL of *E. coli*, *S. aureus* and *GBS* were activated and amplified, and the bacterial solution was mixed at a volume ratio of 1:1:1. A total of 60 mice were randomly divided into control, 3-day, 5-day, and 7-day groups, with 12 mice in each group (3 replicates, n = 4). The control group was infused with PBS transvaginally on day 7 for seven consecutive days; the 3-d group was infused with the bacterial solution on day 11 for three consecutive days; the 5-d group was infused with the bacterial solution on day 9 for five consecutive days; and the 7-d group was infused with the bacterial solution on day 7 for seven consecutive days; all the mice were euthanized on day 15 in order to collect uterine samples, record the body and uterine weights, observe the pathological changes in the tissues, and select the appropriate animal model for the follow-up therapeutic tests.

### 2.5. Treatment of Bacterial Endometritis with Berberine and Carvacrol

#### 2.5.1. Treatment of Bacterial Endometritis in Mice with Berberine and Carvacrol

**Figure 1:**
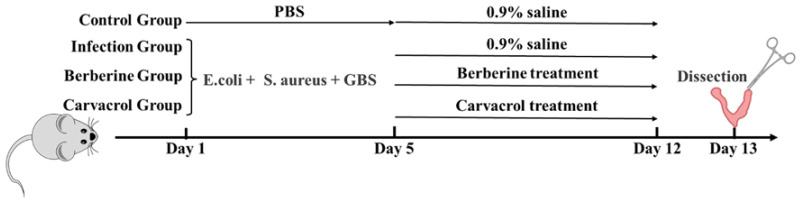
Experimental treatment of endometritis in mice with berberine and carvacrol.

A total of 64 mice were randomly divided into four groups, (n = 16). The experimental manipulations are shown in Figure 1 below. The model, berberine-treated, and carvacrol-treated groups completed modeling on day 5 and began treatment on day 6. The test mice in each group were euthanized on day 13 to collect uterine samples, record body and uterine weights, and observe the pathological changes in the tissues; the samples were then stored at −80 °C.

#### 2.5.2. Measurement of Uterine Index and Histomorphological Observation

The uterus was dissected after euthanasia of the mice; the uterine weight was recorded and the uterine index was calculated (uterine index = uterine weight (mg)/mouse weight (g)). The uterine tissues were fixed in 4% paraformaldehyde and then embedded in paraffin wax and cut into 5 μm sections using a slicer. Subsequently, pathologic evaluation was performed under a light microscope after staining with hematoxylin–eosin (H&E).

#### 2.5.3. Enzyme-Linked Immunosorbent Assay

A total of 50 g of uterine tissue was weighed, and 450 µL of saline was add according to the ratio of weight (g):volume (mL) = 1:9, the tissue was sheared, pre-cooled steel beads were added, and the tissue was broken in a tissue homogenizer to make a 10% homogenate, which was then centrifuged at 2500 r/min for 10 min at 4 °C. The supernatant was remove for the cytokine assay. The levels of pro-inflammatory and anti-inflammatory markers in the supernatant were determined using an enzyme-linked immunosorbent assay kit.

#### 2.5.4. DNA Extraction and Real-Time Fluorescence Quantitative Polymerase Chain Reaction

Total RNA from the uterine tissues was extracted using the Trizol (1 mL/20 mg) method (Beijing Solarbio Science & Technology Co., Ltd., Beijing, China). RNA reverse transcription and RT-qPCR steps were performed according to the manufacturer’s instructions for the experiments. RT-qPCR was run in a LightCycler 480^®^ system (F. Hoffmann-La Roche, Ltd, Basel, Switzerland). Each sample was analyzed in triplicate, and then RT-qPCR was assayed using 2^−∆∆Ct^ relative gene expression analysis, with GAPDH expression as an endogenous control. Primers were synthesized by Sangon Biotech Co., Ltd. (Shanghai, China), and the primer sequences are shown in Table 1. The RT-qPCR reaction program is shown in Table 2.

#### 2.5.5. Western Blotting

RIPA, PMSF, protein phosphatase inhibitor, and inhibitory peptide were mixed in the ratio of 1000:10:10:1 on ice and set aside. A total of 50 mg of uterine tissue was taken into 450 μL of the configured RIPA lysate, which was broken using a homogenizer and centrifuged to obtain protein extracts. After determining the protein concentration of each sample using a BCA assay kit, an equal amount of protein was subjected to 12% SDS-PAGE and then electrotransferred to a PVDF membrane, which was closed with 5% skimmed milk for 1 h at room temperature. The membrane was then shaken with primary antibody overnight at 4 °C and then incubated with secondary antibody for 1 h at room temperature. The blots were detected using an ultrasensitive ECL chemiluminescence kit and visualized using a Bio-Rad imaging system (Bio-Rad, Hercules, CA, USA). Protein quantification, performed with β-actin expression, was normalized. Finally, the resulting bands were analyzed by Image J (Version 1.54h) for gray value.

### 2.6. Statistical Analysis

Data were first evaluated via a Chi-square test using SPSS 21.0 software and then analyzed by one-way ANOVA, with *p* < 0.05 being significant, and *p* < 0.001 being highly significant and the results expressed as SEM ± mean. Finally, bar graphs were plotted using the GraphPad Prism program (Version 10.1.2, GraphPad, Boston, MA, USA).

## 3. Results

### 3.1. In Vitro Antibacterial Activity of Berberine and Carvacrol

Pharmacognostic tests were performed on *Escherichia coli*, *Staphylococcus aureus*, and *Group B Streptococcus* using tablets containing 3.2 g of berberine and carvacrol. The results are presented in Table 3. The diameter of the circle of inhibition of berberine was greater than 10.00 mm, which indicates high sensitivity, and the diameter of the circle of inhibition of carvacrol was greater than 20.00 mm, which indicates super-sensitivity. The minimum bactericidal concentration (MBC) and minimum inhibitory concentration (MIC) of the drug monomers of berberine and carvacrol were explored after multiplicative dilution of the drugs, and the results are shown in Table 4. These results show that berberine and carvacrol displayed significant inhibitory effects against *E. coli*, *S. aureus* and *GBS*. Berberine at a concentration of 4 mg/mL and carvacrol at a concentration of 0.125 mg/mL were selected for the continuation of subsequent trials.

### 3.2. Safety Assessment Results for Berberine and Carvacrol

No corneal damage, iris injury, conjunctival congestion, or conjunctival edema were observed in the eyes of rabbits at any time point, indicating that berberine and carvacrol did not cause significant ocular damage. Additionally, no congestion or edema was observed in the uteri of the mice treated with the drug solution. Compared with the normal group, the endometrium of the mice was structurally intact, the endometrial epithelial cells were neatly arranged, and there was basically no infiltration of inflammatory cells around the endometrium (the results are shown in Figure S1).

### 3.3. Modeling Outcomes of Bacterial Infectious Endometritis

**Figure 2:**
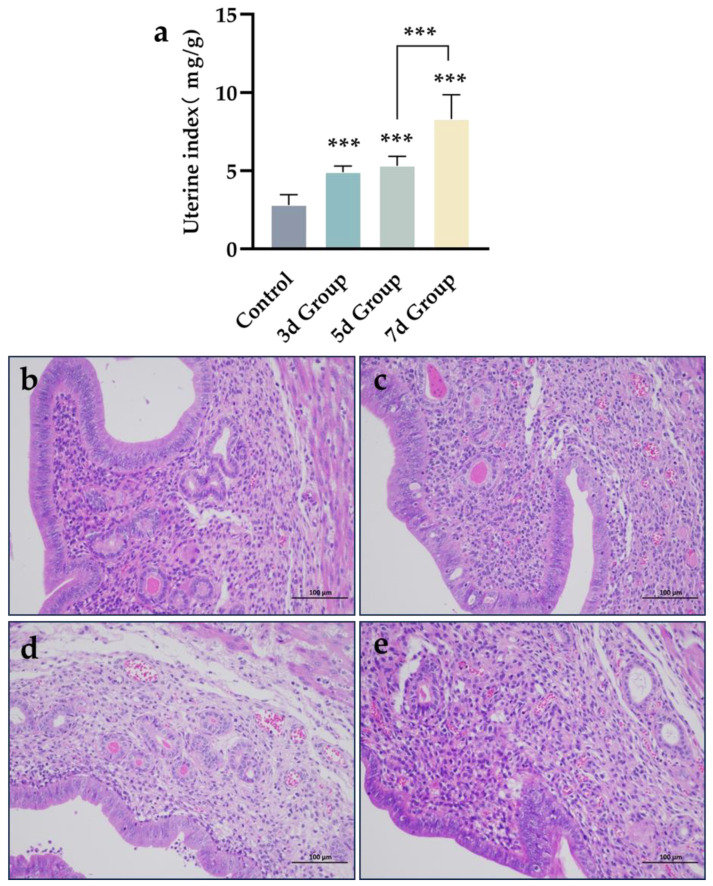
Mixed bacterial fluids cause endometritis in mice. (**a**) Changes in uterine index in mice. (**b**) The control group. (**c**) Bacterial infection after three days. (**d**) Bacterial infection after five days. (**e**) Bacterial infection after seven days. Scale bar is 100 μm. Data statistics are demonstrated as mean ± SEM. *** *p* < 0.001, compared with the control group.

A mixed infection model of endometritis in mice was established using *E. coli*, *S. aureus*, and *GBS*, with the results shown in Figure 2. The uterine index was significantly higher in the 3-day, 5-day and 7-day groups compared to that in the control group (*p* < 0.001); no significant difference was observed between the 3-day and 5-day groups (*p* > 0.05), but the uterine index was significantly higher in the 7-day group compared to that in both the 3-day and 5-day groups (*p* < 0.001). In the control group, the endometrial tissue was structurally intact and exhibited clear morphology. In contrast, in the 3-day and 5-day groups, the connective tissue appeared loosely arranged, the epithelial cells were detached, and there was an increase in inflammatory cells, along with minor hemorrhage. In the 7-day group, the uterine tissue was significantly thinned, and both structural and morphological changes were more pronounced, with severe infiltration of inflammatory cells and more extensive hemorrhage. These results indicate that the mixed bacterial infection successfully induced endometritis in mice, confirming the success of the model.

### 3.4. Therapeutic Efficacy of Berberine and Carvacrol in Treating Bacterial Endometritis

#### 3.4.1. Impact of Berberine and Carvacrol on Uterine Index and Histopathological Changes in Mice

**Figure 3:**
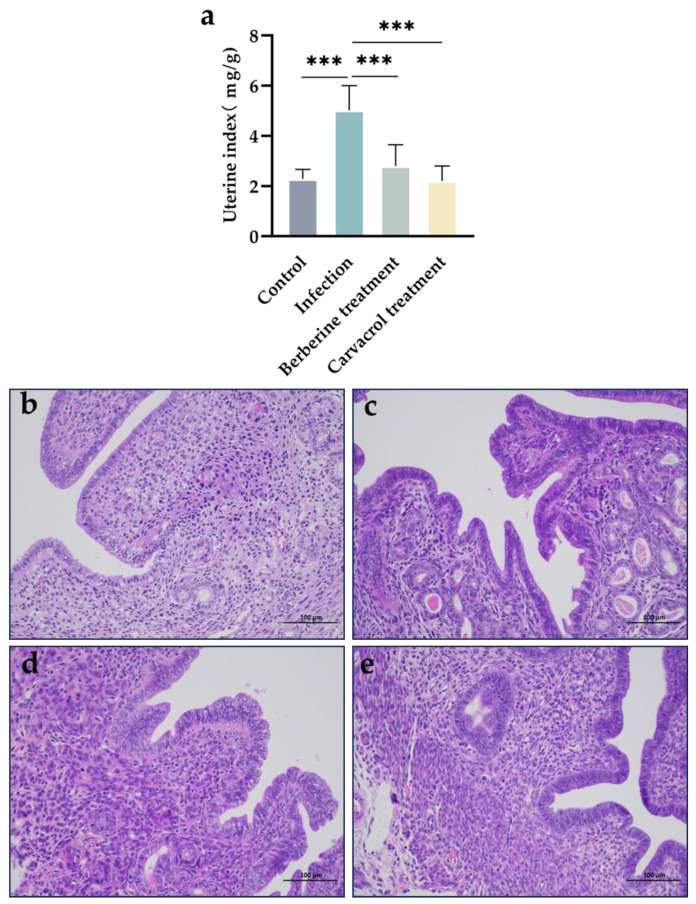
Treatment of endometritis in mice with berberine and carvacrol. (**a**) Changes in uterine index in mice. (**b**) The control group. (**c**) The infection group. (**d**) The berberine treatment group. (**e**) The carvacrol treatment group. Scale bar is 100 μm. Data statistics are demonstrated as mean ± SEM. *** *p* < 0.001, compared with the infection group.

The effects of berberine and carvacrol in the treatment of bacterial-induced endometritis in mice were evident in the histopathological changes observed in the uterus. As shown in Figure 3a, the uterine index was significantly higher in the model group compared to the control group; treatment with both carvacrol and berberine significantly reduced the uterine index, with no significant difference between the treated and control groups (*p* > 0.05). As illustrated in Figure 3b–e, the endometrium in the control group exhibited chorionic protrusion, a complete tissue structure, and clear morphology. In contrast, the endometrium in the model group was invaginated, with loosely arranged connective tissue, detached epithelial cells, increased inflammatory cells, and some hemorrhage; the uterine tissues in the carvacrol and berberine treatment groups were relatively intact and well-preserved. These results suggest that carvacrol and berberine have a reparative effect on uterine tissue damage in mice.

#### 3.4.2. Effects of Berberine and Carvacrol on TLR2 and TLR4 mRNA Expression Levels in the Uterus

**Figure 4:**
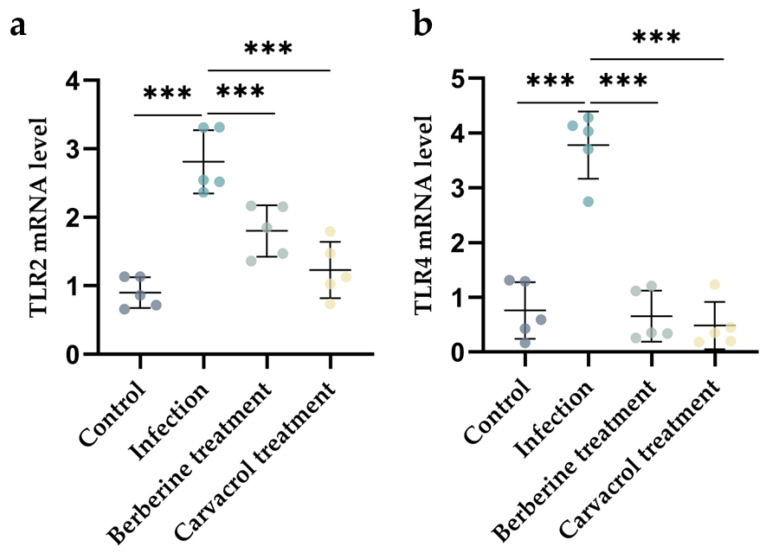
Effects of berberine and carvacrol on TLR2 and TLR4 mRNA expression induced by bacterial infection. (**a**) The relative mRNA expression level of the TLR2 gene. (**b**) The relative mRNA expression level of the TLR4 gene. Data statistics are demonstrated as mean ± SEM. *** *p* < 0.001, compared with the infection group.

TLR2 and TLR4 receptors are defense mechanism receptors of the body against bacterial infection. The relative mRNA expression of TLR2 and TLR4 was detected by RT-qPCR, and the results are shown in Figure 4a,b. The relative mRNA expression of TLR2 and TLR4 in the uterine tissues of mice in the berberine and carvacrol treatment group differed significantly from that of the model group. However, the relative mRNA expression of TLR2 in the berberine treatment group was significantly higher than that in the control group (*p* < 0.01). The test showed that berberine and carvacrol downregulated the mRNA expression of TLR2 and TLR4 in the uterine tissues of endometritis mice. This suggests that berberine and carvacrol treatment inhibited the mRNA expression of TLR2 and TLR4 in bacterial infections.

#### 3.4.3. Effects of Berberine and Carvacrol on the Phosphorylation Levels of Proteins Associated with NF-κB and MAPK Signaling Pathways in the Uterus

**Figure 5:**
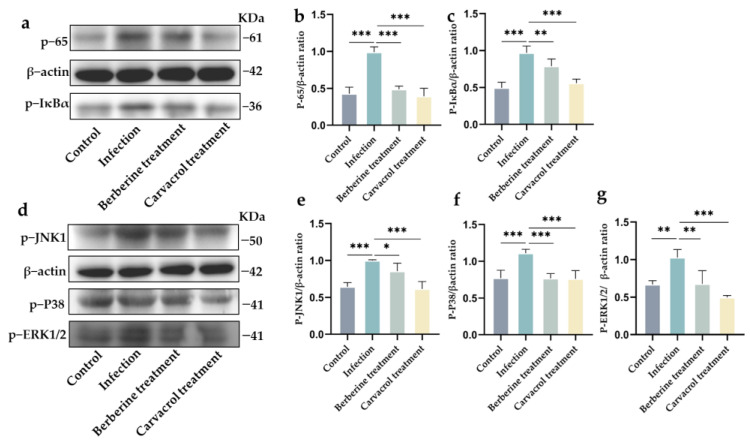
Berberine and carvacrol regulate the phosphorylation levels of NF-κB and MAPK signaling pathway-related proteins in the uterus. (**a**) Western blotting of NF-κB signaling pathway-related proteins. (**b**) Analysis of p-P65 protein gray values. (**c**) Analysis of p-IκBα protein gray values. (**d**) Western blotting of MAPK signaling pathway-related proteins. (**e**) Analysis of p-JNK1 protein gray values. (**f**) Analysis of p-P38 protein gray values. (**g**) Analysis of p-ERK1/2 protein gray values. Data statistics are demonstrated as mean ± SEM. * *p* < 0.05; ** *p* < 0.01; *** *p* < 0.001, compared with the infection group.

By detecting the relative contents of p-P65 and p-IκBα of the NF-κB signaling pathway, as well as p-JNK1, p-P38, and p-ERK1/2 of the MAPK signaling pathway in mouse uterine tissues of the assayed mice (Figure 5), it was found that the relative contents of p-P65, p-IκBα, p-JNK1, p-P38, and p-ERK1/2 were significantly decreased after berberine and carvacrol treatment. After carvacrol treatment, the levels of p-P65, p-IκBα, p-JNK1, p-P38, and p-ERK1/2 were not significantly different from those in the Control group (*p* > 0.05), while after berberine treatment, the levels for p-P65, p-P38, and p-ERK1/2 were not significantly different from those in the control group (*p* > 0.05). These findings suggest that berberine and carvacrol inhibit the phosphorylation of proteins involved in the NF-κB and MAPK signaling pathways in the uterine tissues of mice with endometritis, thereby exerting a mitigating effect on the condition.

#### 3.4.4. Effects of Berberine and Carvacrol on the Expression of Pro- and Anti-Inflammatory Markers Induced by Bacterial Infection

**Figure 6:**
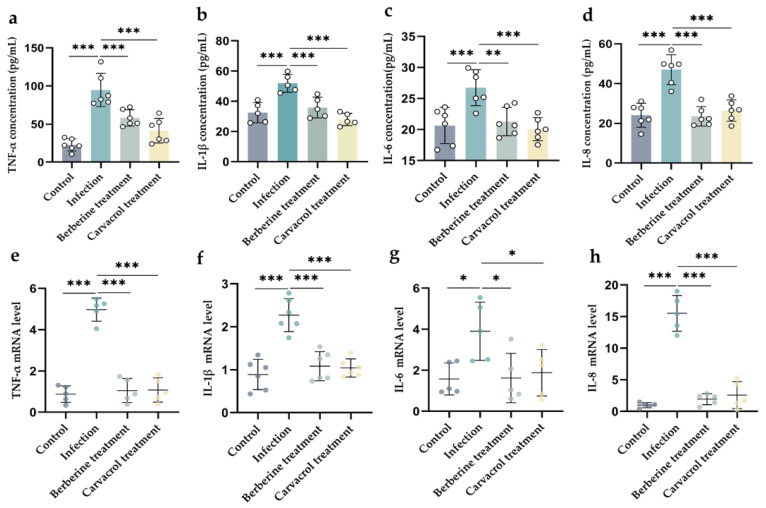
The effect of berberine and carvacrol on cellular infection-induced pro-inflammatory cytokine production. The concentration of (**a**) TNF-a (pg/mL); (**b**) IL-1β (pg/mL); (**c**) IL-6 (pg/mL); (**d**) IL-8 (pg/mL). The relative mRNA expression levels of (**e**) TNF-a; (**f**) IL-1β; (**g**) IL-6; (**h**) IL-8. Data statistics are demonstrated as mean ± SEM. * *p* < 0.05; ** *p* < 0.01; *** *p* < 0.001, compared with the infection group.

Inflammatory cytokines were determined by ELISA. Berberine and carvacrol, respectively, were found to significantly reduce the expression of TNF-a, IL-1β, IL-6, and IL-8 after treatment of endometritis in mice (Figure 6a–d), and the differences were significant compared with those in the model group (*p* < 0.01). The relative expression of inflammatory cytokines TNF-a, IL-1β, IL-6, and IL-8 mRNA in mouse uterine tissues was detected by RT-qPCR, and it was found that the relative expression of inflammatory cytokines TNF-a, IL-1β, and IL-8 mRNA was significantly lower than that in the model group after treatment with berberine and carvacrol (*p* < 0.05), and it was not significantly different from that in the control group (*p* > 0.05) (Figure 6e–h). This indicated that berberine and carvacrol treatment suppressed the expression of relevant inflammatory cytokine genes in uterine tissues of endometritis mice.

## 4. Discussion

Dairy cows are particularly susceptible to metabolic and infectious diseases during the peripartum period. Nutritional deficiencies postpartum can compromise immune function, thereby reducing the cows’ resistance to pathogens [13]. Furthermore, placental remnants and malodor in postpartum cows increase the risk of uterine exposure, promoting microorganism proliferation and subsequently triggering endometritis in dairy cows [14]. Histological examination of the endometrium can detect various cellular components and assess the severity of endometritis. In this study, a mixed bacterial solution was instilled into the mouse uterus transvaginally, resulting in uterine edema and an elevated uterine index. HE staining revealed the infiltration of inflammatory cells in uterine tissues and the detachment of epithelial cells in the uterine mucosa, indicating successful induction of uterine damage. The intrauterine application of antibiotics to inhibit bacterial viability and alleviate endometrial inflammation is the most common clinical treatment [6,15]. However, there are several drawbacks, and its efficacy is influenced by factors such as antibiotic type, severity of endometrial inflammation, and ovarian status [16]. Numerous alternatives to antibiotics for endometritis treatment have been investigated. Intraperitoneal melatonin injection, which promotes autophagy to inhibit mtROS-dependent NLRP3 inflammasome activation, inhibits LPS-induced endometritis and exhibits anti-inflammatory effects [17]. Mesih demonstrated that ozone-enriched ultra-purified water treatment significantly ameliorated E. coli-induced endometrial inflammation after 48 h [18]. Liang modeled endometritis in vitro through dihydrotestosterone treatment. Following LPS-induced inflammation, and reduced expression levels of inflammatory factors IL-1β, IL-6, and TNF-α mRNA, as well as TLR4 and MyD88 proteins in bEECs, were observed [19]. Several of these methods demonstrated efficacy in alleviating endometritis by targeting pathological organization, signaling pathways, and inflammatory factor inhibition, but did not integrate multiple antibacterial and anti-inflammatory approaches. Herbal plant extracts exhibit multiple biological effects, including antibacterial, anti-inflammatory, anticancer, and immunomodulatory properties. Epimedium [20], plant extracts [21], and the Baogong Tang formula [22] have previously been reported to alleviate endometritis, with therapeutic effects. Moreover, Chinese herbs offer several advantages, including high safety, minimal side effects, and the absence of antibiotic residues [23,24]. Data indicate two mechanisms by which Chinese medicines inhibit bacterial growth, i.e., by altering bacterial structure or being metabolized into small molecular compounds that exert therapeutic effects through blood circulation [25]. Chinese medicines not only inhibit bacterial growth but also enhance immunity, inhibit inflammatory cytokine secretion, and mediate cell signaling pathways to reduce inflammation [26]. In this study, in vitro bacteriostatic tests demonstrated that berberine and carvacrol effectively inhibited major pathogenic bacteria, including *Escherichia coli*, *Staphylococcus aureus*, and *Streptococcus agalactiae*, associated with endometritis. Carvacrol at 0.5 mg/mL was found to be more effective in bacterial inhibition [27].

TLR2 and TLR4 are essential receptors involved in bacteria-induced inflammation. Pathogen invasion activates the host’s innate immune response. Pathogen-associated molecular patterns (PAMPs) generated after pathogen infection are specifically recognized by various pattern recognition receptors (PRRs), including Toll-like receptors (TLRs), RIG-I-like receptors (RLRs), and NOD-like receptors (NLRs), which activate innate immune signaling pathways, induce the expression of pro-inflammatory cytokines, and initiate the host’s immune response [28]. The inflammatory pathway activates and recruits neutrophils to generate an inflammatory response that protects uterine tissues; however, an excessive inflammatory response can exacerbate tissue damage. In this study, berberine and carvacrol significantly reduced the mRNA expression of TLR2 and TLR4, inhibited the activation of signaling pathways, and reduced the production of inflammatory cytokines. Previous studies have shown that berberine exhibits significant anti-endotoxin effects and can act as an endotoxin (LPS) antagonist, blocking the LPS/TLR4-induced signaling pathway and inhibiting the resulting inflammatory response [29]. The results of the present study align with previous findings. Upon contact with TLRs, PAMPs activate the MyD88-dependent signaling pathway, which triggers NF-κB and MAPK cascades and induces the expression of genes encoding inflammatory mediators [13]. Numerous inflammatory mediators, including tumor necrosis factor α (TNF-α), and interleukin (IL)-1β, IL-6, and IL-8, are released during endometritis, and inhibition of these inflammatory factors can effectively reduce the severity of bacterial endometritis [1,30,31]. In this experiment, treatment with berberine and carvacrol significantly reduced the phosphorylation levels of p65, IκBα, ERK1/2, JNK1, and p38 and decreased the levels of TNF-α, IL-1β, IL-6, and IL-8, along with the relative expression of their genes in the mouse uterus. These findings are consistent with previous studies where berberine and carvacrol reduced the phosphorylation levels of p65 and IκBα, thereby inhibiting NF-κB pathway activation in LPS-induced mouse mastitis and suppressed MAPK pathway activation by reducing ERK and JNK gene expression, ultimately mitigating tissue damage, oxidative stress, and the inflammatory response [27,32,33,34,35]. These results further validate the therapeutic effects of berberine and carvacrol on endometritis in mice.

Carvacrol has been applied in the treatment of various diseases, including respiratory infections, digestive disorders, and inflammatory conditions, and has also been used as an antimicrobial and antioxidant in studies of natural preservatives in the food industry [36,37]. Berberine is used to treat diseases related to glycolipid metabolism by modulating the microbiota, and it can exhibit anti-inflammatory effects either alone or in combination with other herbal extracts [38]. In this study, treatment with berberine and carvacrol for bacterial endometritis resulted in a relatively intact tissue layer following repair, indicating a potential therapeutic effect on endometritis. However, this study also has some limitations. The etiology of bacterial endometritis is complex and usually involves multiple pathogenic infections, which may affect the efficacy of individual herbal components. The antimicrobial susceptibility of berberine and carvacrol may vary, depending on the particular pathogen. In addition, the hydrophobicity of berberine and berberine, as well as the oxidative properties of carvacrol, pose challenges concerning formulation and drug stability. Therefore, future studies should consider the pathophysiological diversity of endometritis and focus on optimizing herbal-based therapies under different clinical conditions.

## 5. Conclusions

Uterine infusion with berberine (4 mg/mL) and carvacrol (0.125 mg/mL) significantly improved outcomes in a murine model of bacterial-induced endometritis. The mechanism of action lies in the promotion by berberine and carvacrol of uterine tissue repair, reducing TLR2 and TLR4 gene expression, inhibiting NF-κB and MAPK pathway activation, and decreasing pro-inflammatory cytokine levels, indicating their beneficial anti-inflammatory properties.

## Figures and Tables

**Table 1:** Primer information.

Target Gene	Orientations	Primer Sequence (5′-3′)	Lengths	Product Key
TNF-α	F	TCCAGAAGTTGCTTGTGCCT	144	NM_173966.3
	R	CAGAGGGCTGTTGATGGAGG		
IL-1β	F	CCTCGGTTCCATGGGAGATG	119	NM_174093.1
	R	AGGCACTGTTCCTCAGCTTC		
IL-6	F	GCTGAATCTTCCAAAAATGGAGG	215	NM_173923.2
	R	GCTTCAGGATCTGGATCAGTG		
IL-8	F	ACACATTCCACACCTTTCCAC	149	AF232704
	R	ACCTTCTGCACCGACTTTTC		
TLR2	F	TTTGCTCCTGCGAACTCC	109	
	R	GCCACGCCCACATCATTC		
TLR4	F	TATGAACCACTCCACTCGCTC	207	DQ839566
	R	CATCATTTGCTCAGCTCCCAC		
GAPDH	F	GTCTTCACTACCATGGAGAAGG	201	NM_001034034
	R	TCATGGATGACCTTGGCCAG		

**Table 2:** Real-time fluorescence quantitative PCR reaction program.

Reagent Name	Dosages
2 × SuperReal PreMix Plus	10 μL
Forward primer (10 μM)	0.6 μL
Reverse primer (10 μM)	0.6 μL
cDNA templates	2.0 μL
RNase-free ddH_2_O	Replenish to 20 μL

**Table 3:** Circle of inhibition diameters of berberine and carvacrol against *E. coli*, *S. aureus*, and *GBS*.

Drug Name	Drug Concentration (mg/Tablet)	Inhibition Circle Diameter (mm)		
		*E. coli*	*S. aureus*	*GBS*
Control group	0	6.00 ± 0.00	6.00 ± 0.00	6.00 ± 0.00
Berberine group	3.2	16.36 ± 0.12	13.50 ± 0.60	15.40 ± 0.29
Carvacrol group	3.2	51.36 ± 0.86	46.90 ± 0.76	23.97 ± 0.32

**Table 4:** MIC and MBC of berberine and carvacrol against *E. coli*, *S. aureus*, and *GBS*.

Bacterial Species	Drug Treatment (mg/mL)			
	Berberine		Carvacrol	
	MIC	MBC	MIC	MBC
*E. coli*	8	2	0.5	0.5
*S. aureus*	8	2	0.5	0.0625
*GBS*	16	4	0.25	0.0625

## Data Availability

Data supporting the findings are available from the corresponding author upon reasonable request.

## Supplementary Materials

The following supporting information can be downloaded at: https://www.mdpi.com/article/10.3390/microorganisms13051051/s1, Figure S1: Effects of drug accumulation on the mouse uterus. (a) Control group. (b) Berberine group. (c) Carvacrol group.

## Abbreviations

*E. coli*: *Escherichia coli*; *S. aureus*, *Staphylococcus aureus*; *GBS*, *Streptococcus agalactiae*; HE staining, hematoxylin–eosin staining; RT-PCR, reverse transcription-polymerase chain reaction; ELISA, enzyme-linked immunosorbent assay; mRNA, messenger RNA; TLR2, Toll-like receptor 2; TLR4, Toll-like receptor 4; NF-κB, nuclear factor kappa-B; MAPK, mitogen-activated protein kinase; PGF2α, prostaglandin F2alphα; TNF-α, tumor necrosis factor-α; IL-1β, interleukin-1 β; ERK 1/2, extracellular regulated protein kinases 1/2; LPS, lipopolysaccharide; EIF2AK2, eukaryotic translation initiation factor 2-alpha kinase 2; IL-18β, interleukin-18 β; IL-2, interleukin-2; MBC, minimum bactericidal concentration; MIC, minimum inhibitory concentration; p-P65, phosphorylated P65 protein; p-P38, phosphorylated P38 protein.
