# Supplementary material for: Mechanistic Study on the Alleviation of Endometritis in Mice Through Inhibition of NF-κB and MAPK Signaling Pathways by Berberine and Carvacrol
Source: Microorganisms. 2025 Apr 30;13(5):1051. doi: 10.3390/microorganisms13051051 (PMC12114256; doi:10.3390/microorganisms13051051)
Supplement: Supplementary file 1 [file microorganisms-13-01051-s001.zip › microorganisms-3521258-supplementary.pdf]

microorganisms

MDPI

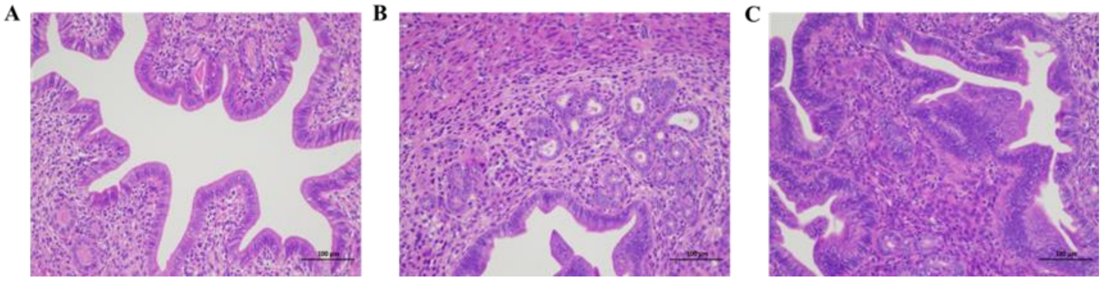

Figure S1. Histopathologic sections of uterine tissues (HE, ×100). Control group (A); chaparral group (B); carvacrol group (C).
